# Association between meal context and meal quality: an ecological momentary assessment in Japanese adults

**DOI:** 10.1007/s00394-024-03416-9

**Published:** 2024-05-03

**Authors:** Nana Shinozaki, Kentaro Murakami, Nana Kimoto, Shizuko Masayasu, Satoshi Sasaki

**Affiliations:** 1 Department of Social and Preventive Medicine, School of Public Health, The University of Tokyo, 7-3-1 Hongo, Bunkyo-ku, Tokyo, 113-0033 Japan; 2 Ikurien-naka, Sugaya, Naka-shi, Ibaraki 3799-6, 311–0105 Japan

**Keywords:** Meal context, Diet quality, Ecological momentary assessment, Situational factors, Japanese, Dietary record

## Abstract

**Purpose:**

The purpose was to assess the relationship between the quality of meals and its context.

**Methods:**

We conducted a cross-sectional study of 222 Japanese adults aged 30–76 years in 2021. The following information was obtained from the 4-d weighed dietary records: the recording day (working or not), meal type (breakfast, lunch, or dinner), eating companions (alone or with someone), eating location (at home or away from home), and screen-based activity (yes or no). The nutritional quality of each meal was evaluated using the Healthy Eating Index 2020 (HEI-2020).

**Results:**

The analysis included 1,295 meals for males and 1,317 for females. The mean HEI-2020 ranged from 43.0 (lunch) to 51.9 (dinner) in males and from 45.7 (breakfast) to 52.0 (dinner) in females. Multilevel linear regression showed that, in males, lunch had a significantly lower HEI-2020 score compared to breakfast (*β* = −1.81, 95% confidence interval [CI]: −3.42, − 0.20), while dinner had a significantly higher HEI-2020 score (*β* = 6.77, 95% CI: 5.34, 8.20). Eating with someone was significantly associated with a higher HEI-2020 score (*β* = 2.22, 95% CI: 0.76, 3.67). Among females, dinner had a higher HEI-2020 score than breakfast (*β* = 5.21, 95% CI: 3.72, 6.70). Eating away from home was associated with higher HEI-2020 scores (*β* = 2.14, 95% CI: 0.04, 4.24).

**Conclusion:**

Meal type, location, and eating companions were associated with meal quality in this population, with differences between males and females. Incorporating these factors in nutrition education and interventions can enhance diet quality.

**Supplementary Information:**

The online version contains supplementary material available at 10.1007/s00394-024-03416-9.

## Introduction

Poor diet quality has been considered as a risk factor for all-cause mortality, cardiovascular disease, cancer, and type 2 diabetes [1]. Although the Japanese diet is generally perceived as healthier than other diets [2], diet quality in Japan is not as high as expected. In a nationally representative sample of Japanese adults, the mean total score of the Healthy Eating Index-2015, a widely accepted diet quality index, was similar to that of Americans [3]. Thus, there is an urgent need to improve dieta quality, and understanding the factors affecting diet quality is crucial for developing effective interventions to improve public health.

Meal context, such as where, with whom, and in what environment food is consumed, has been recognised as an important determinant of diet quality [4–6]. For instance, available evidence suggests that eating out is associated with an unfavourable dietary intake characterised by a high intake of energy, total fat, and sugars [7–9]. In addition, media use at mealtime [10] and eating dinner with others [11] were associated with lower fruit and vegetable intake. However, previous studies have exclusively focused on identifying the contextual factors contributing to between-individual differences, and little is known about the factors related to within-individual variations in dietary behaviour [12]. Furthermore, previous research has used questionnaires to measure meal contexts [11, 13, 14], which do not provide a detailed description of meal contexts on eating occasions [15]. As contextual factors are dynamic and can fluctuate momentarily, identifying momentary contextual factors associated with within-person variations in dietary intake is important for understanding and improving dietary behaviours [16].

In recent years, the ecological momentary assessment (EMA), which repeatedly samples participants’ current behaviours and experiences in their natural environments in real-time, has been increasingly used in nutritional research [17–19]. EMA can help reduce recall bias, maximise ecological validity (i.e. generalisability to people’s daily lives and the natural environment), and capture experience and behaviour over time and across settings within individuals in real-world contexts [17–19]. The EMA has been used to explore contextual factors related to the healthiness of a diet [15, 20–29], offering a novel framework for studying the determinants of diet quality. However, most studies have focused on selected foods (e.g. fruits and vegetables) [15, 20–26] or specific meal types (e.g. dinner) [27, 28], and only one study has evaluated the associations between various meal contexts and the overall diet quality of meals across different meal types [29]. In addition, because previous studies included only youth with type 1 diabetes mellitus [29], the associations between diet quality and meal contexts should be evaluated in populations with broader participant characteristics.

Therefore, in this study of healthy Japanese adults, we aimed to examine the associations between the overall diet quality of meals and meal context, specifically focusing on meal type (breakfast, lunch, and dinner), eating companions (alone or with someone), eating location (at home or away from home), and screen-based activities (yes or no). We utilised the EMA technique based on detailed dietary data collected from dietary records (DRs) to capture the within-individual level phenomenon. We hypothesised that several contextual factors were associated with meal quality.

## Materials and methods

### Study participants and procedure

This cross-sectional study was based on data from a validation study of an online diet history questionnaire administered between August and October 2021. Details of the study procedures and participants have been described previously [30]. Briefly, research dietitians (n 66) with experience in DR surveys collected data from 14 of the 47 prefectures. In each prefecture, eight cohabiting couples, consisting of two females each in four age categories (30–39, 40–49, 50–59, and 60–69 years) and their husbands (regardless of age) were recruited, resulting in 112 females and 112 males. The sample size was determined based on the purpose of the original validation study [30]. The inclusion criterion was free-living individuals with Internet access. The exclusion criteria were dietitians, people living with a dietitian, people receiving dietary guidance from a doctor or dietitian, people receiving insulin therapy or dialysis treatment, people who had difficulty completing web questionnaires, people with special dietary habits such as avoiding commonly consumed food for religious or health reasons, and pregnant and breastfeeding women. Because of snowball sampling, the number of people who approached and were excluded from the survey was not recorded. This study is reported according to the adapted STROBE Checklist for Reporting EMA Studies [31].

### Dietary record

A total of 111 females aged 30–69 years and 111 males aged 30–76 years completed a 4-non-consecutive-day weighed DR after a couple withdrew. The details of the DR have been previously described [30]. Briefly, the recording days were three weekdays (Monday through Friday) and one weekend day (Saturday or Sunday) for two weeks. Each couple was instructed by the assigned research dietitian, both verbally and in writing. They were given an example of a completed diary sheet and asked to record all foods and beverages they consumed on the provided recording sheet and weigh them using a provided digital cooking scale (KS-274, Dretec, Japan; ±2 g accuracy for 0–500 g, ± 3 g accuracy for 500–2000 g). When weighing was difficult, participants were asked to provide as much information as possible about the food, including the name of the products, brands, or restaurants and the approximate amount consumed or leftover.

The research dietitians retrieved the recording sheets immediately after each DR was completed. The research dietitian checked the content of the records and asked the participants for additional information in person, by phone or email, as needed. The weights of food reported in approximate quantities were estimated as accurately as possible based on information from general recipes, websites, or product labels. Each food was assigned a food code based on the 2015 version of the Standard Tables of Food Composition of Japan [32] using a standard procedure. The recording sheets were sent to the study’s central office and rechecked by trained dieticians.

### Nutritional quality of each meal

The DR sheets consisted of sections on breakfast, lunch, dinner, and snacks. In this study, meal types (breakfast, lunch, dinner, and snacks) were determined based on the section in which the meal was written. Snacks were excluded from the main analysis because of our focus on the quality of the three main meals, considering the relatively low frequency of snacks [33] and their low contribution to the total energy and nutrient intake [34] among Japanese adults. The number of meals was 1,295 (425 breakfast, 426 lunch, and 444 dinner) for males and 1,317 (439 breakfast, 436 lunch, and 442 dinner) for females.

The energy and nutrient content of each meal were estimated based on the weight of the food items and their nutrient content using the Standard Tables of Food Composition of Japan [32]. As the database lacks the amount of added sugar for some foods, the added sugar content for such foods was calculated based on the same or similar foods in the 2011–2012 Food Patterns Equivalents Database [35]. Teaspoon equivalents in the Food Patterns Equivalents Database were converted into grams by multiplying with 4.2 (grams of added sugar per teaspoon). The mean daily total energy intake (including snacks) was calculated for each individual.

The nutritional quality of each meal assessed using the Healthy Eating Index 2020 (HEI-2020) [36]. The components and scoring criteria of the HEI-2020 are completely aligned with those of the HEI-2015 [37], which has been used to assess the quality of the total diet [38] and individual meals [34, 39]. The usefulness of the HEI-2020 in Japanese has been previously verified [3]. The HEI-2020 is a 100-point scale used to evaluate adherence to the 2020–2025 Dietary Guidelines for Americans [40], with higher scores indicating better diet quality. The nine adequacy components (maximum score) are total fruits (5), whole fruits (5), total vegetables (5), greens and beans (5), wholegrains (5), dairy (10), total protein foods (5), seafood and plant proteins (5) and fatty acids (the ratio of the sum of polyunsaturated and monounsaturated fatty acids to saturated fatty acids) (10), and the four moderation components include refined grains (10), sodium (10), added sugars (10) and saturated fats (10). We calculated the HEI-2020 score for each meal based on energy-adjusted values (i.e. amount/1000 kcal of energy or percentage of energy) using the 2011–2012 Food Patterns Equivalents Database [40]. The HEI-2020 total and component scores were also calculated for each individual for each meal and overall diet (including snacks) based on the average intake over the four-day period.

### Meal context

On each recording day, the participants recorded whether the day was (i) a working or school day, (ii) a non-working or non-school day, or (iii) other schedules (e.g. non-working or non-school days for retirees) on the DR sheet. Because of the low frequency of ‘other schedules’, we combined ‘non-working or non-school days’ and ‘other schedules’ for the analysis. In addition, for each eating occasion, participants were asked to record where they ate (at home, workplace, restaurant, or other places), the number of people they ate with, and whether they ate while watching a monitor, such as TV, computers, or mobile phones. Because the number of people included in each category of the place to eat and the number of people they ate together were unbalanced, the eating location was reclassified into home or away from home, and eating companions were grouped into alone (i.e. ate alone) or with someone (i.e. ate with one or more people).

### Basic characteristics

The basic characteristics of the participants (sex, age, body height, body weight, education level, and current smoking status) were collected using web questionnaires. Body mass index (BMI) was calculated as weight (kg) divided by height squared (m^2^). Educational level was grouped into three categories (junior high school or high school, junior college or technical school, and university or higher). Smoking status was reported as smoking more than 20 cigarettes per day, 20 cigarettes or less per day, past smoking, or never smoking, and was grouped into ‘current smoker’ and ‘never or former smoker’.

The accuracy of reported energy intake (EI) was assessed using Goldberg’s cut-off for the ratio of EI to basal metabolic rate (BMR) [41]. Participants were categorised as either plausible, under-reporters, or over-reporters of EI based on whether their ratio fell within, below, or above the 95% confidence limits for agreement between EI: BMR and their corresponding physical activity level (PAL). The PAL for sedentary lifestyle (1.55) was used for all participants in this study due to the absence of an objective measure of physical activity. The BMR was calculated using an equation designed for Japanese individuals, which considers height, weight, age, and sex [42, 43].

We calculated the upper and lower cut-off values for the agreement between EI: BMR and PAL with 95% confidence limits. This considered the coefficient of variation in intakes and other components of energy balance. Specifically, we considered the within-subject variation in EI at 23%, the precision of the estimated BMR relative to the measured BMR at 8.5%, and the between-subject variation in PAL at 15% [41]. Based on this, we defined under-reporters, plausible reporters, and over-reporters as those with an EI: BMR of less than 1.02, between 1.02 and less than 2.35, and 2.35 or greater, respectively [41].

### Statistical analysis

Statistical analyses were performed using SAS version 9.4 (SAS Institute Inc., Cary, NC, US). Our sample of cohabiting couples was analysed separately according to sex (males and females) [30]. This a priori decision was made because of marked differences in diet quality [44] and eating behaviours [45] between sexes, which may be diluted by combined analyses of cohabiting couples consuming similar diets. Statistical significance was defined as a two-sided *P*-value of < 0.05. Descriptive statistics are reported as means and standard deviations (SDs) or numbers and percentages. The Kolmogorov-Smirnov tests were used to assess the normality of the distribution of individual HEI-2020 scores; an unpaired t-test was used to assess sex differences in HEI-2020 total scores. Moreover, to compare means of HEI-2020 total and component scores between meals, multiple comparisons with Bonferroni correction were used.

Multilevel linear modelling was used to account for multiple observations (meals) within each participant. We employed random intercepts and an unstructured covariance matrix with multiple eating occasions (Level 1) nested within individuals (Level 2). The dependent variable was the HEI-2020 score for each meal as the continuous variable. The independent variables included meal context variables (level-1 predictors) and participants’ basic characteristics (level-2 predictors). Level-1 predictors included meal type (breakfast, lunch, or dinner), day type (working or non-working day), eating location (at home or away from home), eating companions (alone or with someone), and screen-based activities while eating (yes or no). Level-2 predictors consisted of age (years, continuous), BMI kg/m^2^, continuous), education level (junior high school or high school, college or technical school, or university or higher), smoking status (never/former smoker or current smoker), and EI (kcal/d, continuous). All categorical independent variables were dummy-coded. Level-1 variables were group-mean centred, whereas level-2 variables were grand-mean centred for appropriate interpretation of the model parameters [46, 47]. The sample size for each sex was sufficiently large to obtain unbiased estimates of Level 2 standard errors [48]. A power calculation was conducted using G∗Power 3.1.9.7, assuming a small effect size of 0.1, four days of dietary recordings with three meals per day, an intraclass correlation coefficient of 0.5, and a two-tailed a of 0.05 [49]. The calculation showed that a sample size of 111 participants for each sex yielded a statistical power of 0.96.

In this study, a step-by-step approach was used to build multilevel linear models. The independent variables at each level were added to the model step by step using PROC MIXED procedure [50, 51]. First, an intercept-only model (model 0) with no independent variables was used to calculate intraclass correlations (ICCs) and design effects [46]. Non-zero ICCs (0.19 for males and 0.21 for females) and design effects greater than 2.0 (3.0 for males and 3.3 for females) indicated variance in the HEI scores of meals across individuals, showing the need for multilevel analysis [46]. Next, Model 1 was fitted to the level-1 variables to evaluate the association between meal context factors and meal quality. Model 2 was then fitted to level-2 variables to evaluate the association between individual-level factors and meal quality, and Model 3 was fitted for both level-1 and level-2 eating factors. The equations for these models are presented in Text S1.

The fixed effects of the independent variables were evaluated using regression coefficients with 95% confidence intervals (CIs). The regression coefficients and variances were estimated using maximum likelihood, as the regression coefficients are of major interest [50]. Akaike’s information criterion (AIC) was used to assess the goodness of fit of each model [46]. Multicollinearity among the independent variables was checked using the variance inflation factor (VIF) in the final model. None of the variables showed multicollinearity problems (VIF < 5) [52]. Sensitivity analyses were performed to evaluate the association between meal quality and meal contextual factors after excluding under- and over-reporters of EI.

## Results

### Characteristics of the study participants and eating occasions

Table 1 presents the basic characteristics of the study participants. The mean age was 51.7 years (SD: 11.9) for males and 49.9 years (SD: 10.7) for females. The mean BMI was 23.8 kg/m^2^ (SD: 3.6) for males and 22.7 kg/m^2^ (SD: 3.3) for females. The most common educational level was university or higher for males (43.2%) and junior college or technical school for females (49.6%). More than two-thirds of males and females were either never smokers or former smokers. There were no over-reporters of EI; over 90% of the participants were classified as plausible reporters of EI, while the remaining individuals were under-reporters. The mean HEI-2020 score for overall diet was higher in females than males (51.6 vs. 49.4, *P* = 0.01). Compared to breakfast and lunch, dinner was significantly high in HEI-2020 total score and component scores for total vegetables, total protein foods, seafood and plant proteins, and refined grains in both sexes (Table S1).

**Table 1:** Basic characteristics of the study participants^1^

Variables	Male (*n* 111)	Female (*n* 111)
Age (years)	51.7 ± 11.9	49.9 ± 10.7
Body height (cm)	170.2 ± 6.3	158.4 ± 5.4
Body weight (kg)	68.9 ± 11.9	56.9 ± 8.5
Body mass index (kg/m^2^)	23.8 ± 3.6	22.7 ± 3.3
Education level, n (%)		
Junior high school or high school	41 (36.9)	28 (25.2)
Junior college or technical school	22 (19.8)	55 (49.6)
University or higher	48 (43.2)	28 (25.2)
Smoking status, n (%)		
Never or former smoker	76 (68.5)	99 (89.2)
Current smoker	35 (31.5)	12 (10.8)
Energy intake (kcal/d)	2067 ± 446	1552 ± 301
Energy reporting status^2^, n (%)		
Underreporting	11 (9.9)	10 (9.0)
Plausible reporting	100 (90.1)	101 (91.0)
Overreporting	0 (0)	0 (0)
HEI-2020 score (maximum: 100 points)		
Breakfast	43.3 ± 12.4	45.7 ± 13.0
Lunch	43.0 ± 9.8	46.5 ± 9.7
Dinner	51.9 ± 8.2	52.0 ± 8.0
Overall diet (including snacks)	49.4 ± 9.2	51.6 ± 9.0

HEI, Healthy Eating Index

^1^Values are means ± standard deviations unless otherwise indicated

^2^Underreporting, plausible reporting, and overreporting were defined as participants having a ratio of reported energy intake to BMR of < 1.02, ≥ 1.02 to < 2.35, and ≥ 2.35, respectively

Table 2 shows the meal context characteristics of the eating occasions. Meals were evenly distributed among breakfast, lunch, and dinner for both sexes. Males had a higher percentage of eating occasions on working or school days (64.3%), whereas females had a higher percentage of eating occasions on non-working days or non-school days (53.7%). The most common eating behaviours among both male and female participants were consuming meals at home, with someone else, and not engaging in screen-based activities.

**Table 2:** Meal context characteristics (n 1295 for males and n 1317 for females)

		Male		Female	
		N	%	N	%
Meal type					
	Breakfast	425	32.8	439	33.3
	Lunch	426	32.9	436	33.1
	Dinner	444	34.3	442	33.6
Day type					
	Working or school day	832	64.3	610	46.3
	Non-working day or non-school day	463	35.8	707	53.7
Eating location					
	At home	987	76.2	1095	83.1
	Away from home	308	23.8	222	16.9
Eating companion					
	Alone	556	42.9	394	29.9
	With someone	739	57.1	923	70.1
Screen-based activity while eating					
	No	857	66.2	897	68.1
	Yes (watching TV, computer, or mobile phone)	438	33.8	420	31.9

### Factors associated with meal quality

Table 3 shows the associations between meal context, individual characteristics, and HEI-2020 meal scores among males. The ICC value of the null model (Model 0) indicated that 19% of the variance in the HEI-2020 scores of meals was explained by between-individual variability, leaving 81% of the variance in the HEI-2020 scores explained by within-individual variability on different eating occasions. The fixed effects of each variable observed in Models 1 and 2 were also observed in Model 3, in the same direction and magnitude. Model 3 showed a lower AIC score than Models 1 and 2, indicating a better goodness-of-fit. The final model revealed that compared to breakfast, lunch had a significantly lower HEI-2020 score (*β* = 1.81, 95% CI: −3.42, − 0.20), while dinner had a significantly higher HEI-2020 score (*β* = 6.77, 95% CI: 5.34, 8.20). Eating with someone was associated with a higher HEI-2020 score (*β* = 2.22, 95% CI: 0.76, 3.67). Regarding individual-level factors, age was positively associated with the HEI-2020 score, and current smokers had a lower HEI-2020 score than never or former smokers.

**Table 3:** Factors associated with the HEI-2020 score at 1295 eating occasions in 111 males

			Model 0 (null model)	Model 1 (eating occasion-level variables)	Model 2 (individual-level variables)	Model 3 (eating occasion- and individual-level variables)
**Fixed effect**						
**Intercept**			43.25 (42.11, 44.39)^***^	43.25 (42.11, 44.39)^***^	43.28 (42.38, 44.18)^***^	43.28 (42.38, 44.19)^***^
**Eating occasion-level variables**						
	Meal type	Breakfast	-	Ref	-	Ref
		Lunch	-	-1.81 (-3.42, -0.20)^*^	-	-1.81 (-3.42, -0.20)^*^
		Dinner	-	6.77 (5.34, 8.20)^***^	-	6.77 (5.34, 8.20)^***^
	Day type	Working or school day	-	Ref	-	Ref
		Non-working day or non-school day	-	-0.20 (-2.17, 1.77)	-	-0.20 (-2.17, 1.77)
	Eating location	At home	-	Ref	-	Ref
		Away from home	-	1.89 (-0.05, 3.82)	-	1.89 (-0.05, 3.82)
	Eating companion	Alone	-	Ref	-	Ref
		With someone	-	2.22 (0.76, 3.67)^**^	-	2.22 (0.76, 3.67)^**^
	Screen-based activity while eating	No	-	Ref	-	Ref
		Yes (watching TV, computer, or mobile phone)	-	-0.49 (-2.11, 1.12)	-	-0.49 (-2.11, 1.12)
**Individual-level variables**						
	Age (years)		-	-	0.19 (0.11, 0.28)^***^	0.19 (0.11, 0.28)^***^
	BMI (kg/m^2^)		-	-	-0.12 (-0.39, 0.14)	-0.12 (-0.38, 0.14)
	Energy intake (kcal/d)				0.004 (0.002, 0.006)^***^	0.004 (0.002, 0.006)^***^
	Education level	Junior high school or high school	-	-	Ref	Ref
		College or technical school	-	-	-1.00 (-3.65, 1.64)	-1.00 (-3.64, 1.65)
		University or higher	-	-	0.39 (-1.73, 2.51)	0.40 (-1.72, 2.52)
	Smoking status	Never or former smoker	-	-	Ref	Ref
		Current smoker	-	-	-4.13 (-6.24, -2.02)^***^	-4.13 (-6.23, -2.02)^***^
**Variance components (random effects)**						
	Level-2 Intercept		26.68^***^	28.05^***^	12.90^***^	14.30^***^
	Residual		117.41^***^	101.22^***^	117.45^***^	101.25^***^
**Model Summary**						
	AIC		9996.1	9832.4	9956.4	9792.8
	ICC		0.19	0.22	0.10	0.12

AIC, Akaike information criterion; BMI, body mass index; CI, confidence interval; ICC, intraclass correlation coefficient; Ref, reference category; HEI, Healthy Eating Index

The dependent variable was the HEI score for meals (maximum: 100 points). Values for eating occasion-level and individual-level variables show regression coefficients with 95% confidence intervals in parentheses. Other values are parameter estimates, with 95% confidence intervals in parentheses, if any. The regression coefficients represent the change in the HEI-2020 score for a one-unit increase for age and BMI and the difference in HEI-2020 scores compared to the reference category for other independent variables.

^*^P < 0.05, ^**^P < 0.01, ^***^P < 0.001.

Table 4 presents the results of the same analysis for females. According to the ICC value for the null model, 21% of the variance in the HEI-2020 scores of meals was explained by between-individual variability, and the remaining 79% of the variance in the HEI-2020 score was explained by within-individual variability on different eating occasions. Model 3 showed similar associations of meal context variables and individual characteristics with HEI-2020 scores, as in Models 1 and 2. In the final model, dinner had a significantly higher HEI-2020 score than breakfast (*β* = 5.21, 95% CI: 3.72, 6.70). Eating away from home was associated with a higher HEI-2020 score (*β* = 2.14, 95% CI: 0.04, 4.24). Regarding individual-level factors, age and BMI were positively associated with the HEI-2020 score, and current smokers had lower HEI-2020 scores than never or former smokers.

**Table 4:** Factors associated with the HEI-2020 score at 1317 eating occasions in 111 females

			Model 0 (null model)	Model 1 (eating occasion-level variables)	Model 2 (individual-level variables)	Model 3 (eating occasion- and individual-level variables)
**Fixed effect**						
**Intercept**			44.33 (43.12, 45.55)^***^	44.33 (43.12, 45.55)^***^	44.33 (43.21, 45.46)^***^	44.33 (43.21, 45.46)^***^
**Eating occasion-level variables**						
	Meal type	Breakfast	-	Ref	-	Ref
		Lunch	-	-1.36 (-3.00, 0.27)	-	-1.36 (-3.00, 0.27)
		Dinner	-	5.21 (3.72, 6.70)^***^	-	5.21 (3.72, 6.70)^***^
	Day type	Working or school day	-	Ref	-	Ref
		Non-working day or non-school day	-	-1.18 (-3.26, 0.89)	-	-1.18 (-3.26, 0.89)
	Eating location	At home	-	Ref	-	Ref
		Away from home	-	2.14 (0.04, 4.24)^*^	-	2.14 (0.04, 4.24)^*^
	Eating companion	Alone	-	Ref	-	Ref
		With someone	-	-0.57 (-2.08, 0.95)	-	-0.57 (-2.08, 0.95)
	Screen-based activity while eating	No	-	Ref	-	Ref
		Yes (watching TV, computer, or mobile phone)	-	-0.34 (-2.06, 1.39)	-	-0.34 (-2.06, 1.39)
**Individual-level variables**						
	Age (years)		-	-	0.11 (0, 0.22)	0.11 (0, 0.22)^*^
	BMI (kg/m^2^)		-	-	0.37 (0.02, 0.73)^*^	0.37 (0.02, 0.73)^*^
	Energy intake (kcal/d)				0.001 (-0.002, 0.005)	0.001 (-0.002, 0.005)
	Education level	Junior high school or high school	-	-	Ref	Ref
		College or technical school	-	-	0.81 (-2.03, 3.64)	0.80 (-2.03, 3.64)
		University or higher	-	-	2.92 (-0.35, 6.20)	2.92 (-0.35, 6.19)
	Smoking status	Never or former smoker	-	-	Ref	Ref
		Current smoker	-	-	-5.49 (-9.26, -1.72)^**^	-5.49 (-9.26, -1.71)^**^
**Variance components (random effects)**						
	Level-2 Intercept		31.67^***^	32.28^***^	25.65^***^	26.26^***^
	Residual		119.13^***^	111.89^***^	119.14^***^	111.9^***^
**Model Summary**						
	AIC		10197.1	10115.4	10173.9	10098.2
	ICC		0.21	0.22	0.18	0.19

AIC, Akaike information criterion; BMI, body mass index; CI, confidence interval; ICC, intraclass correlation coefficient; Ref, reference category; HEI, Healthy Eating Index

The dependent variable was the HEI score for meals (maximum: 100 points). Values for eating occasion-level and individual-level variables show regression coefficients with 95% confidence intervals in parentheses. Other values are parameter estimates, with 95% confidence intervals in parentheses, if any. The regression coefficients represent the change in the HEI-2020 score for a one-unit increase for age and BMI and the difference in HEI-2020 scores compared to the reference category for other independent variables.

*P < 0.05, **P < 0.01, ***P < 0.001.

Tables S1 and S2 show the results of the sensitivity analysis, which included only plausible reporters of EI. The findings were largely consistent with the main analysis, except that BMI was no longer associated with the HEI-2020 score among females.

## Discussion

### Main findings

To the best of our knowledge, this is the first study to examine the association between the overall diet quality of meals and various meal contexts in adults using EMA. In this population, meal type, location, and eating companions were found to be associated with meal quality, and these factors differed between males and females. Specifically, compared to breakfast, lunch showed a lower diet quality among males, and dinner showed a higher diet quality among males and females. Moreover, eating with someone for males and eating away from home for females was associated with better meal quality. Regarding individual characteristics, younger age and current smoking were associated with lower meal quality in both sexes.

### Comparison with previous studies

Dinner has been reported to have the highest nutritional quality among all meal types in the Japanese population [34, 39]. The results of the present study were similar, indicating that dinner is independently associated with higher nutritional quality, even after considering other contextual factors (e.g. eating with others) and individual characteristics. Previous studies on the Japanese diet have shown that dinner contributed the most to the total EI [34, 39, 53] and was the longest of the three main meals [54]. Furthermore, compared to breakfast and lunch, dinner has a different combination of foods [39] and dishes [55], particularly characterised by a high vegetable intake [34, 39, 53]. In the present study, dinner had high intakes of total vegetables, total protein foods, seafood and plant proteins and low refined grain intake. Therefore, dinners may be considered the predominant meal of the day in Japan, with nutrient-rich main or side dishes consisting of vegetables and protein sources. In contrast, a previous study in the US showed that the nutritional quality of breakfast and dinner was comparable in youths with type 1 diabetes [29]. This discrepancy may be attributed to differences in eating habits and population characteristics. It is also unclear why the nutritional quality of lunch was lower than that of breakfast among males in this population. Given the paucity of studies, further research is needed to clarify the association between the nutritional quality of meals and meal type.

A previous study on young Australian adults found no association between meal quality and social interaction (e.g. with family members and friends) during mealtime [20]. Similarly, among African American females, eating with others was not associated with the consumption of snacks or sugar-sweetened beverages [16]. In this study, eating with someone was associated with higher meal quality only in males. It is well known that eating with others influences food choices and portions, which may be partly explained by the modelling of dietary behaviour (i.e. adjusting food intake to that of eating companions) [56–58]. Thus, the meal quality of the male participants in this study may have been positively affected by eating companions with better diet quality. Given that only cohabiting couples were included in this study and that females had better overall diet quality than males, it is possible that eating with one’s wife may have positively impacted males’ meal quality. However, since information on who was with the participants during the meal was unavailable in this study, further research is needed to examine the association between the nutritional quality of meals and the presence of others.

Previous reviews have suggested that eating out is associated with poorer diet quality, characterised by a higher intake of energy, total and saturated fats, sugars, and sodium, and a lower intake of fibre, dairy, fruit, vegetables, and key micronutrients [7, 9]. This is also supported by several previous studies; when eating outside the home, Italian college students had a lower probability of consuming vegetables, fruits, and legumes, and a higher probability of consuming processed meats, salty snacks, and alcoholic beverages than when eating at home [25]. In addition, among young Australian individuals, eating in a café or restaurant, rather than at home, was associated with higher sugar-sweetened beverage consumption [22]. Interestingly, our results showed that females reported better meal quality when eating away from home. Among this population, the workplace was the most common eating location for meals away from home, accounting for 70% and 78% of meals for males and females, respectively. As this study focused on the places where meals were eaten, workplace meals included boxed lunches prepared at home and brought to work. Given that food prepared at home is more likely to contain nutritious foods, such as fruits and vegetables [20, 26], collecting information on where meals are prepared may provide more insight into the association between meal quality and eating out.

No association was observed between meal quality and mealtime screening-based activities. This is consistent with the results of several studies [6, 20]. In contrast, other studies have reported negative associations between meal healthiness and mealtime screen-based activities, such as watching TV, in children [15] and adults [10, 16, 28]. Furthermore, meal quality was not associated with whether the day was a workday. Prior research on the association between whether it is a workday and meal quality is limited [59]. Further research is needed to understand the relationship between meal quality, screen-based activities, and workdays.

### Feature implication

This study showed that the healthiness of meals is influenced by various factors, including where, when, and with whom one eats, as well as by individual characteristics, such as age and smoking status. Therefore, future public health interventions should target not only personal characteristics but also contextual factors, such as developing behavioural strategies for specific eating situations [6, 12]. For example, implementing policies that help males eat healthy meals during lunchtime or when eating alone can improve overall diet quality and lead to significant public health benefits. In addition, inconsistencies between the present and previous studies regarding the relationship between meal quality and some meal context variables, suggest that the determinants of food intake during each eating occasion may not be generalisable across different countries [22]. Hence, further studies are warranted to investigate this aspect.

### Strengths and limitations

A strength of this study was the use of a 4-day DR. Detailed information about dietary intake and eating situations repeatedly measured within individuals allowed us to conduct an EMA for the association between meal quality and meal contexts. Moreover, the DR period included weekdays and weekends, enabling us to capture changes in dietary behaviour and meal environments depending on the daily schedule. Nevertheless, this study has several limitations. First, although recruitment was conducted across a wide area in Japan, the study participants were not randomly selected but rather volunteers who may be more health-conscious than the general population. The educational level of the present population was higher than that of a nationally representative sample of males (junior high school or high school: 52.9%; junior college or technical school: 12.9%; university or higher: 33.7%) and females (55.9%, 27.6%, 15.6%, respectively) [60]. However, the percentage of current smokers and the means (SDs) of height, weight and BMI in this population were similar to those in a nationally representative sample (males: 27.1%, 167.7 (6.9) cm, 67.4 (12.0) kg and 23.9 (3.6) kg/m^2^, respectively; females: 7.6%, 154.3 (6.7) cm, 53.6 (9.2) kg and 22.5 (3.7) kg/m^2^, respectively) [61]. In addition, because this study only included cohabiting couples, the findings cannot be applied to people with other living conditions (e.g. living alone). Therefore, further investigations should be conducted using a more representative sample of individuals from various backgrounds. Second, because of the cross-sectional design, it was impossible to determine a causal relationship between meal context and meal quality. For instance, in the case of males, it was unclear whether eating with others increases meal quality or if they do not eat with others because the quality of their meals is low. Therefore, further interventional studies are needed to draw clear conclusions. Third, detailed information was not captured for some meal context variables. For example, there was no information on who was with the participant at mealtime (e.g. family, friends, coworkers), which could also be related to diet quality [15]. Additional contextual factors, such as the location of preparing and purchasing foods [20, 22], cognition, mood, and physiological state [12, 18], may also affect the nutritional quality of each meal. In addition, individual characteristics, such as cooking and food skill confidence [62], are potential factors related to diet quality. Therefore, future studies should examine more detailed and broader meal contexts and participant characteristics. Fourth, snacks were not examined in this study. Although most Japanese adults consume snacks infrequently and in small amounts [33, 34], some individuals may consume them more frequently, which could impact their overall dietary intake. To gain a better understanding of how meal context affects the nutritional quality of snacks, larger studies that include snacks should be conducted in the future. Fifth, because the weighed DR is susceptible to measurement errors [63], the diet quality may have been inaccurate. When weighing was difficult, the consumed weight of foods was estimated, which may have resulted in inaccurate estimates of meal quality. Moreover, it is possible that some participants changed their dietary behaviour by recording their diet [63]. However, it is challenging to identify measurement errors at the meal level. Nonetheless, we assessed the quality of the diet by using energy-adjusted values, which can mitigate measurement errors. We also verified that the association between meal context and quality remained consistent even after eliminating individuals who underreported their EI. Finally, four days of DRs may be insufficient to capture habitual dietary behaviours. Recording dietary intake and meal context using mobile device-based technologies such as mobile apps [22, 25, 64] or wearable cameras [20] would effectively extend the recording period while reducing the burden on the participants. However, it should be noted that in addition to the high cost of developing the equipment and software [17, 31], image coding is time-consuming [20].

## Conclusion

This study found that the nutritional quality of lunch was lower for males compared to breakfast, whereas the nutritional quality of dinner was higher for both sexes. In addition, eating with someone was associated with higher meal quality in males, whereas eating away from home was linked to better meal quality in females. These findings using EMA techniques shed light on the barriers and facilitators of healthy eating in real-world settings and can inform the development of more effective public health interventions. However, further research is needed to gain a better understand of the relationship between meal context and quality.

## Electronic supplementary material

Below is the link to the electronic supplementary material.

Supplementary Material 1

## Data Availability

The datasets generated and analysed during the present study are not publicly available due to privacy and ethical restrictions imposed by the Ethics Committee of the University of Tokyo, Faculty of Medicine, but are available from the corresponding author upon reasonable request.

## Abbreviations

AIC: Akaike’s information criterion
BMI: body mass index
BMR: basal metabolic rate
CI: confidence interval
DR: dietary record
EI: energy intake
EMA: ecological momentary assessment
HEI: Healthy Eating Index
ICC: intraclass correlation
VIF: variance inflation factor
