# Supplementary material for: Association between meal context and meal quality: an ecological momentary assessment in Japanese adults
Source: Eur J Nutr. 2024 May 3;63(6):2081–93. doi: 10.1007/s00394-024-03416-9 (PMC11377557; doi:10.1007/s00394-024-03416-9)
Supplement: Supplementary file 1 — Supplementary Material 1 [file 394_2024_3416_MOESM1_ESM.docx]

**Association between meal context and meal quality: an ecological momentary assessment in Japanese adults**

European Journal of Nutrition

Nana Shinozaki^1^, Kentaro Murakami^1*^, Nana Kimoto^1^, Shizuko Masayasu^2^, and Satoshi Sasaki^1^

^1^ Department of Social and Preventive Medicine, School of Public Health, The University of Tokyo, 7-3-1 Hongo, Bunkyo-ku, Tokyo 113-0033, Japan

^2^ Ikurien-naka, 3799-6 Sugaya, Naka-shi, Ibaraki 311–0105, Japan

*Corresponding author: Dr. Kentaro Murakami, E-mail: kenmrkm@m.u-tokyo.ac.jp

**Text S1: Model equations for the mixed effects regression models**

HEI: Healthy eating index

**Model 0:**

Y_ij_ = γ_00_ + u_0j_ + r_ij_

Y_ij_: the HEI-2020 score of meal *i* in participant *j*

γ_00_: a grand-mean HEI-2020 score of all participants

u_0j_: a participant-specific deviation in HEI-2020 scores from the grand mean.

r_ij_ : a meal-specific deviation in HEI-2020 scores from the mean HEI-2020 score of participant *j*.

**Model 1:**

Y_ij_ = γ_00_ + β_1_Day type+β_2_Meal type+β_3_Eating location+β_4_Eating companion +β_5_Screen-based activity while eating+ u_0j_+ r_ij_

Y_ij_: the HEI-2020 score of meal *i* in participant *j*

γ_00_: a grand-mean HEI-2020 score

β_1_-β_4_: the average effect of each independent variable on the HEI-2020 at each eating occasion score across all participants

u_0j_: a participant-specific deviation in HEI-2020 scores from the grand mean.

r_ij_ : a meal-specific deviation in HEI-2020 scores from the mean HEI-2020 score of participant *j*.

Independent variables were dummy-coded and group-mean centred.

**Model 2:**

Y_ij_ = γ_00_ + β_1_Age+β_2_Body mass index+β_3_Education level+β_4_Smoking status +β_5_Daily Energy intake+ u_0j_+ r_ij_

Y_ij_: the HEI-2020 score of meal *i* in participant *j*

γ_00_: a grand-mean HEI-2020 score

β_1_-β_5_: the average effect of each independent variable on the HEI-2020 at each eating occasion score across all participants

u_0j_: a participant-specific deviation in HEI-2020 scores from the grand mean.

r_ij_ : a meal-specific deviation in HEI-2020 scores from the mean HEI-2020 score of participant *j*.

Dummy variables were created for education level and smoking status categories. Independent variables were dummy-coded and group-mean centred.

**Model 3:**

Y_ij_ = γ_00_ +β_1_Day type+β_2_Meal type+β_3_Eating location+β_4_Eating companion +β_5_Screen-based activity while eating+ β_6_Age+β_7_Body mass index+β_8_Education level+β_9_Smoking status +β_10_Daily Energy intake+ u_0j_+ r_ij_

Y_ij_: the HEI-2020 score of meal *i* in participant *j*

γ_00_: a grand-mean HEI-2020 score

β_1_-β_5_: the average effect of each independent variable on the HEI-2020 at each eating occasion score across all participants

u_0j_: a participant-specific deviation in HEI-2020 scores from the grand mean.

r_ij_ : a meal-specific deviation in HEI-2020 scores from the mean HEI-2020 score of participant *j*.

Dummy variables were created for categorical variables. Level-1 independent variables (variables entered into Model 1) were group-mean centred, and Level-2 independent variables (variables entered into Model 2) were grand-mean centred.

| Table S1. Mean estimates of the total and component scores of Healthy Eating Index-2020 (HEI-2020) derived from 4-d dietary records in 222 Japanese males and females, according to meal type^1^ | | | | | | | | | | | | | | | | | | | |
| --- | --- | --- | --- | --- | --- | --- | --- | --- | --- | --- | --- | --- | --- | --- | --- | --- | --- | --- | --- |
|  | Male (n 111) | | | | | | | | |  | Female (n 111) | | | | | | | | |
|  | Breakfast | | | Lunch | | | Dinner | | |  | Breakfast | | | Lunch | | | Dinner | | |
| HEI-2020^2^ | Mean | | SD | Mean | | SD | Mean | | SD |  | Mean | | SD | Mean | | SD | Mean | | SD |
| Total score (100)^3^ | 43.3 | ^a^ | 12.4 | 43.0 | ^a^ | 9.8 | 51.9 | ^b^ | 8.2 |  | 45.7 | ^a^ | 13.0 | 46.5 | ^a^ | 9.7 | 52.0 | ^b^ | 8.0 |
| Total fruits (5) | 1.5 | ^a^ | 2.0 | 0.6 | ^b^ | 1.2 | 0.5 | ^b^ | 1.0 |  | 2.1 | ^a^ | 2.2 | 1.1 | ^b^ | 1.6 | 0.7 | ^b^ | 1.3 |
| Whole fruits (5) | 1.9 | ^a^ | 2.3 | 0.8 | ^b^ | 1.6 | 0.8 | ^b^ | 1.5 |  | 2.5 | ^a^ | 2.4 | 1.7 | ^b^ | 2.0 | 1.1 | ^b^ | 1.7 |
| Total vegetables (5) | 2.2 | ^a^ | 2.1 | 3.5 | ^b^ | 1.4 | 4.7 | ^c^ | 0.7 |  | 2.4 | ^a^ | 2.0 | 3.9 | ^b^ | 1.3 | 4.8 | ^c^ | 0.7 |
| Greens and beans (5) | 0.9 | ^a^ | 1.7 | 1.2 | ^a^ | 1.7 | 1.9 | ^b^ | 2.0 |  | 1.1 | ^a^ | 1.9 | 1.6 | ^ab^ | 2.0 | 2.1 | ^b^ | 2.1 |
| Whole grains (10) | 0.3 | ^a^ | 1.4 | 0.8 | ^b^ | 2.4 | 0.4 | ^ab^ | 1.6 |  | 0.8 |  | 2.2 | 0.8 |  | 2.5 | 0.5 |  | 1.9 |
| Dairy (10) | 3.5 | ^a^ | 4.0 | 1.0 | ^b^ | 1.9 | 0.9 | ^b^ | 1.4 |  | 4.6 | ^a^ | 4.0 | 1.7 | ^b^ | 2.5 | 0.8 | ^b^ | 1.2 |
| Total protein foods (5) | 3.5 | ^a^ | 1.9 | 4.1 | ^b^ | 1.3 | 4.9 | ^c^ | 0.3 |  | 3.3 | ^a^ | 2.0 | 4.2 | ^b^ | 1.1 | 4.9 | ^c^ | 0.3 |
| Seafood and plant proteins (5) | 2.9 | ^a^ | 2.3 | 3.1 | ^a^ | 1.9 | 4.7 | ^b^ | 1.1 |  | 2.9 | ^a^ | 2.3 | 3.6 | ^b^ | 1.8 | 4.6 | ^c^ | 1.3 |
| Fatty acids (10)^4^ | 4.5 | ^a^ | 3.9 | 7.0 | ^b^ | 3.2 | 7.8 | ^b^ | 2.6 |  | 4.0 | ^a^ | 3.8 | 6.9 | ^b^ | 3.3 | 8.0 | ^c^ | 2.6 |
| Refined grains (10) | 2.0 | ^a^ | 3.6 | 0.4 | ^b^ | 1.4 | 4.4 | ^c^ | 4.1 |  | 2.0 | ^a^ | 3.4 | 1.1 | ^b^ | 2.3 | 4.9 | ^c^ | 3.9 |
| Sodium (10) | 4.7 | ^a^ | 3.9 | 2.1 | ^b^ | 3.2 | 2.7 | ^b^ | 3.2 |  | 4.9 | ^a^ | 4.1 | 2.0 | ^b^ | 3.2 | 1.7 | ^b^ | 2.8 |
| Saturated fats (10) | 8.3 | ^a^ | 3.0 | 9.6 | ^b^ | 1.1 | 9.8 | ^b^ | 0.9 |  | 8.7 | ^a^ | 2.3 | 9.5 | ^b^ | 1.0 | 9.7 | ^b^ | 1.2 |
| Added sugars (10) | 7.1 | ^a^ | 3.3 | 8.9 | ^b^ | 2.1 | 8.5 | ^b^ | 2.3 |  | 6.5 | ^a^ | 3.7 | 8.4 | ^b^ | 2.4 | 8.2 | ^b^ | 2.3 |
| SD, standard deviation. ^1^Means in a row with a different superscript letter significantly differ (*P*<0.05 with Bonferroni adjustments for multiple comparisons). ^2^The maximum scores are shown in parentheses. A higher score indicates higher diet quality. ^3^Calculated as the sum of all components scores.  ^4^Defined as the ratio of the sum of PUFA and MUFA to SFA. | | | | | | | | | | | | | | | | | | | |

| Table S2. Factors associated with the HEI-2020 score at 1169 eating occasions in 100 male plausible reporters of energy intake^1^ | | | | | | |
| --- | --- | --- | --- | --- | --- | --- |
|  |  |  | **Model 0**  (null model) | **Model 1**  (eating occasion-level variables) | **Model 2** (individual-level variables) | **Model 3** (eating occasion- and individual-level variables) |
| **Fixed effect** | |  |  |  |  |  |
| **Intercept** | |  | 43.48 (42.24, 44.72)^***^ | 43.48 (42.24, 44.72)^***^ | 43.51 (42.54, 44.48)^***^ | 43.51 (42.55, 44.49)^***^ |
| **Eating occasion-level variables** | |  |  |  |  |  |
|  | Meal type | Breakfast | - | Ref | - | Ref |
|  |  | Lunch | - | -1.70 (-3.39, -0.01)^*^ | - | -1.70 (-3.39, -0.01)^*^ |
|  |  | Dinner | - | 6.91 (5.40, 8.23)^***^ | - | 6.91 (5.40, 8.43)^***^ |
|  | Day type | Working or school day | - | Ref | - | Ref |
|  |  | Non-working day or non-school day | - | -0.14 (-2.25, 1.97) | - | -0.14 (-2.25, 1.97) |
|  | Eating location | At home | - | Ref | - | Ref |
|  |  | Away from home | - | 1.24 (-0.80, 3.28) | - | 1.24 (-0.80, 3.29) |
|  | Eating companion | Alone | - | Ref | - | Ref |
|  |  | With someone | - | 1.96 (0.41, 3.52)^*^ | - | 1.96 (0.41, 3.52)^*^ |
|  | Screen-based activity while eating | No | - | Ref | - | Ref |
|  |  | Yes (watching TV, computer, or mobile phone) | - | -0.50 (-2.22, 1.22) | - | -0.50 (-2.22, 1.22) |
| **Individual-level variables** | |  |  |  |  |  |
|  | Age (years) |  | - | - | 0.20 (0.11, 0.29)^***^ | 0.20 (0.11, 0.29)^***^ |
|  | BMI (kg/m^2^) |  | - | - | -0.19 (-0.48, 0.09) | -0.19 (-0.48, 0.09) |
|  | Energy intake (kcal) |  |  |  | 0.004 (0.002, 0.007)^***^ | 0.004 (0.002, 0.007)^***^ |
|  | Education level | Junior high school or high school | - | - | Ref | Ref |
|  |  | College or technical school | - | - | -1.74 (-4.62, 1.15) | -1.73 (-4.62, 1.15) |
|  |  | University or higher | - | - | 0.42 (-1.79, 2.63) | 0.42 (-1.79, 2.63) |
|  | Smoking status | Never or former smoker | - | - | Ref | Ref |
|  |  | Current smoker | - | - | -4.33 (-6.57, -2.08)^***^ | -4.33 (-6.58, -2.08)^***^ |
| **Variance components (random effects)** | | |  |  |  |  |
|  | Level-2 Intercept |  | 28.72^***^ | 30.16^***^ | 13.63^***^ | 15.09^***^ |
|  | Residual |  | 119.13^***^ | 102.27^***^ | 119.16^***^ | 102.30^***^ |
| **Model Summary** | |  |  |  |  |  |
|  | AIC |  | 9045.3 | 8917.6 | 9008.6 | 8857.6 |
|  | ICC |  | 0.19 | 0.23 | 0.10 | 0.13 |
| AIC, Akaike information criterion; BMI, body mass index; CI, confidence interval; ICC, intraclass correlation coefficient; Ref, reference category; HEI, Healthy Eating Index | | | | | | |
| The dependent variable was the HEI score for meals (maximum: 100 points). Values for eating occasion-level and individual-level variables show regression coefficients with 95% confidence intervals in parentheses. Other values are parameter estimates, with 95% confidence intervals in parentheses, if any. The regression coefficients represent the change in the HEI-2020 score for a one-unit increase for age and BMI and the difference in HEI-2020 scores compared to the reference category for other independent variables. | | | | | | |
| ^*^*P*< 0.05, ^**^*P*< 0.01, ^***^*P*< 0.001. | | | | | | |
| ^1^ Plausible reporting was defined as participants having a ratio of reported energy intake to basal metabolic rate of ≥1.02 to <2.35. | | | | | | |

| Table S3. Factors associated with the HEI-2020 score at 1200 eating occasions in 101 female plausible reporters of energy intake | | | | | | |  |
| --- | --- | --- | --- | --- | --- | --- | --- |
|  |  |  | **Model 0**  (null model) | **Model 1**  (eating occasion-level variables) | **Model 2** (individual-level variables) | **Model 3** (eating occasion- and individual-level variables) |  |
| **Fixed effect** | |  |  |  |  |  |  |
| **Intercept** | |  | 44.25 (42.97, 45.52)^***^ | 44.25 (42.97, 45.51)^***^ | 44.24 (43.07, 45.42)^***^ | 44.24 (43.07, 45.42)^***^ |  |
| **Eating occasion-level variables** | |  |  |  |  |  |  |
|  | Meal type | Breakfast | - | Ref | - | Ref |  |
|  |  | Lunch | - | -1.13 (-2.79, 0.53) | - | -1.13 (-2.79, 0.54) |  |
|  |  | Dinner | - | 5.35 (3.82, 6.88)^***^ | - | 5.35 (3.82, 6.88)^***^ |  |
|  | Day type | Working or school day | - | Ref | - | Ref |  |
|  |  | Non-working day or non-school day | - | -0.88 (-2.97, 1.21) | - | -0.88 (-2.97, 1.21) |  |
|  | Eating location | At home | - | Ref | - | Ref |  |
|  |  | Away from home | - | 2.57 (0.42, 4.73)^*^ | - | -2.57 (-0.42, 4.73) |  |
|  | Eating companion | Alone | - | Ref | - | Ref |  |
|  |  | With someone | - | -0.70 (-2.27, 0.86) | - | -0.70 (-2.27, 0.86) |  |
|  | Screen-based activity while eating | No | - | Ref | - | Ref |  |
|  |  | Yes (watching TV, computer, or mobile phone) | - | -0.10 (-1.69, 1.89) | - | -0.10 (-1.69, 1.89) |  |
| **Individual-level variables** | |  |  |  |  |  |  |
|  | Age (years) |  | - | - | 0.15 (0.03, 0.27)^*^ | 0.15 (0.03, 0.27)^*^ |  |
|  | BMI (kg/m^2^) |  | - | - | 0.27 (-0.11, 0.65) | 0.27 (-0.11, 0.65) |  |
|  | Energy intake (kcal) |  |  |  | 0.003 (-0.002, 0.008) | 0.001 (-0.002, 0.008) |  |
|  | Education level | Junior high school or high school | - | - | Ref | Ref |  |
|  |  | College or technical school | - | - | 0.65 (-2.30, 3.60) | 0.65 (-2.30, 3.60) |  |
|  |  | University or higher | - | - | 2.47 (-0.99, 5.92) | 2.47 (-0.99, 5.92) |  |
|  | Smoking status | Never or former smoker | - | - | Ref | Ref |  |
|  |  | Current smoker | - | - | -5.24 (-9.57, -0.91)^*^ | -5.24 (-9.57, -0.91)^**^ |  |
| **Variance components (random effects)** | | |  |  |  |  |  |
|  | Level-2 Intercept |  | 31.83^***^ | 32.42^***^ | 25.74^***^ | 26.26^***^ |  |
|  | Residual |  | 115.01^***^ | 107.94^***^ | 115.01^***^ | 111.9^***^ |  |
| **Model Summary** | |  |  |  |  |  |  |
|  | AIC |  | 9252.4 | 9194.7 | 9248.5 | 10098.2 |  |
|  | ICC |  | 0.22 | 0.23 | 0.18 | 0.19 |  |
| AIC, Akaike information criterion; BMI, body mass index; CI, confidence interval; ICC, intraclass correlation coefficient; Ref, reference category; HEI, Healthy Eating Index | | | | | | | |
| The dependent variable was the HEI score for meals (maximum: 100 points). Values for eating occasion-level and individual-level variables show regression coefficients with 95% confidence intervals in parentheses. Other values are parameter estimates, with 95% confidence intervals in parentheses, if any. The regression coefficients represent the change in the HEI-2020 score for a one-unit increase for age and BMI and the difference in HEI-2020 scores compared to the reference category for other independent variables. | | | | | | |  |
| *P< 0.05, **P< 0.01, ***P< 0.001. | | | | | | |  |
| ^1^ Plausible reporting was defined as participants having a ratio of reported energy intake to basal metabolic rate of ≥1.02 to <2.35. | | | | | | |  |
